# Salt-Enrichment Impact on Biomass Production in a Natural Population of Peatland Dwelling Arcellinida and Euglyphida (Testate Amoebae)

**DOI:** 10.1007/s00248-018-1296-8

**Published:** 2018-12-11

**Authors:** Alex Whittle, Matthew J. Amesbury, Dan J. Charman, Dominic A. Hodgson, Bianca B. Perren, Stephen J. Roberts, Angela V. Gallego-Sala

**Affiliations:** 1 Department of Geography, University of Exeter, Exeter, EX4 4RJ UK; 2 British Antarctic Survey, Natural Environment Research Council, High Cross, Madingley Road, Cambridge, CB3 0ET UK; 3 Environmental Change Research Unit (ECRU), Faculty of Biological and Environmental Sciences, University of Helsinki, Helsinki, Finland; 4 Department of Geography, Durham University, Durham, DH1 3LE UK

**Keywords:** Testate amoebae, Sub-Antarctica, Salinity, Southern hemisphere westerly winds, Bioindicators

## Abstract

Unicellular free-living microbial eukaryotes of the order Arcellinida (Tubulinea; Amoebozoa) and Euglyphida (Cercozoa; SAR), commonly termed testate amoebae, colonise almost every freshwater ecosystem on Earth. Patterns in the distribution and productivity of these organisms are strongly linked to abiotic conditions—particularly moisture availability and temperature—however, the ecological impacts of changes in salinity remain poorly documented. Here, we examine how variable salt concentrations affect a natural community of Arcellinida and Euglyphida on a freshwater sub-Antarctic peatland. We principally report that deposition of wind-blown oceanic salt-spray aerosols onto the peatland surface corresponds to a strong reduction in biomass and to an alteration in the taxonomic composition of communities in favour of generalist taxa. Our results suggest novel applications of this response as a sensitive tool to monitor salinisation of coastal soils and to detect salinity changes within peatland palaeoclimate archives. Specifically, we suggest that these relationships could be used to reconstruct millennial scale variability in salt-spray deposition—a proxy for changes in wind-conditions—from sub-fossil communities of Arcellinida and Euglyphida preserved in exposed coastal peatlands.

**Electronic supplementary material:**

The online version of this article (10.1007/s00248-018-1296-8) contains supplementary material, which is available to authorized users.

Arcellinida and Euglyphida (AE) belong to a polyphyletic group of test-forming protozoans that are widely used in environmental biomonitoring and palaeoclimatology [1, 2]. Despite the predominant association of most taxa with freshwater ecosystems (e.g. lakes, soils, peatlands) the distribution of active communities also extends into many moderately saline environments [3–8]. Nevertheless, like other microbial groups [9, 10], physiological and metabolic challenges exerted by differing salinity conditions appear to be reflected in the global and local distribution of AE. Only a small percentage of taxa are known to occur in marine ecosystems [e.g. 11], and salinity conditions have been linked to patterns in the distribution of taxa within lakes contaminated by road salt run-off [12, 13], anchialine pools [14], and saltmarshes [3, 15, 16]. However, the importance of salinity in defining the distribution of taxa, community structure, and size of AE populations remains uncertain.

We directly examined changes in a population of AE along an uninterrupted salinity gradient from brackish to freshwater conditions within a west-facing coastal peatland on sub-Antarctic Marion Island (Fig. 1). Correlation between salt-enrichment and coastal exposure is a characteristic feature of ecosystems on the sub-Antarctic islands which results from intense generation and deposition of oceanic salt-spray by strong prevailing westerly winds [17]. Soil-dwelling AE communities were described from 28 samples exposed to varying levels of relative salt-enrichment which was quantified by measuring the conductivity of pore-water, a direct proxy for microhabitat salinity (Fig. 1; Supplementary methods). Principal component (PC) analysis confirmed that conductivity was the primary ecological gradient represented by the samples (Fig. 2a) and suggested that atmospheric deposition of oceanic salt-spray was the dominant cause of elevated salinity (see Supplementary methods). The peatland transect, which spanned a coastal terrace ~ 30 m above sea-level, represented a model system for assessing the ecological importance of salinity because; (1) the carbon and moisture rich substrate provides consistent conditions for colonisation, (2) confounding micro-environmental conditions thought to affect the distribution of AE in other salt-influenced systems were controlled, and (3) the low-energy depositional environment limits potential for contamination of assemblages by allochthonous tests.

**Fig. 1:**
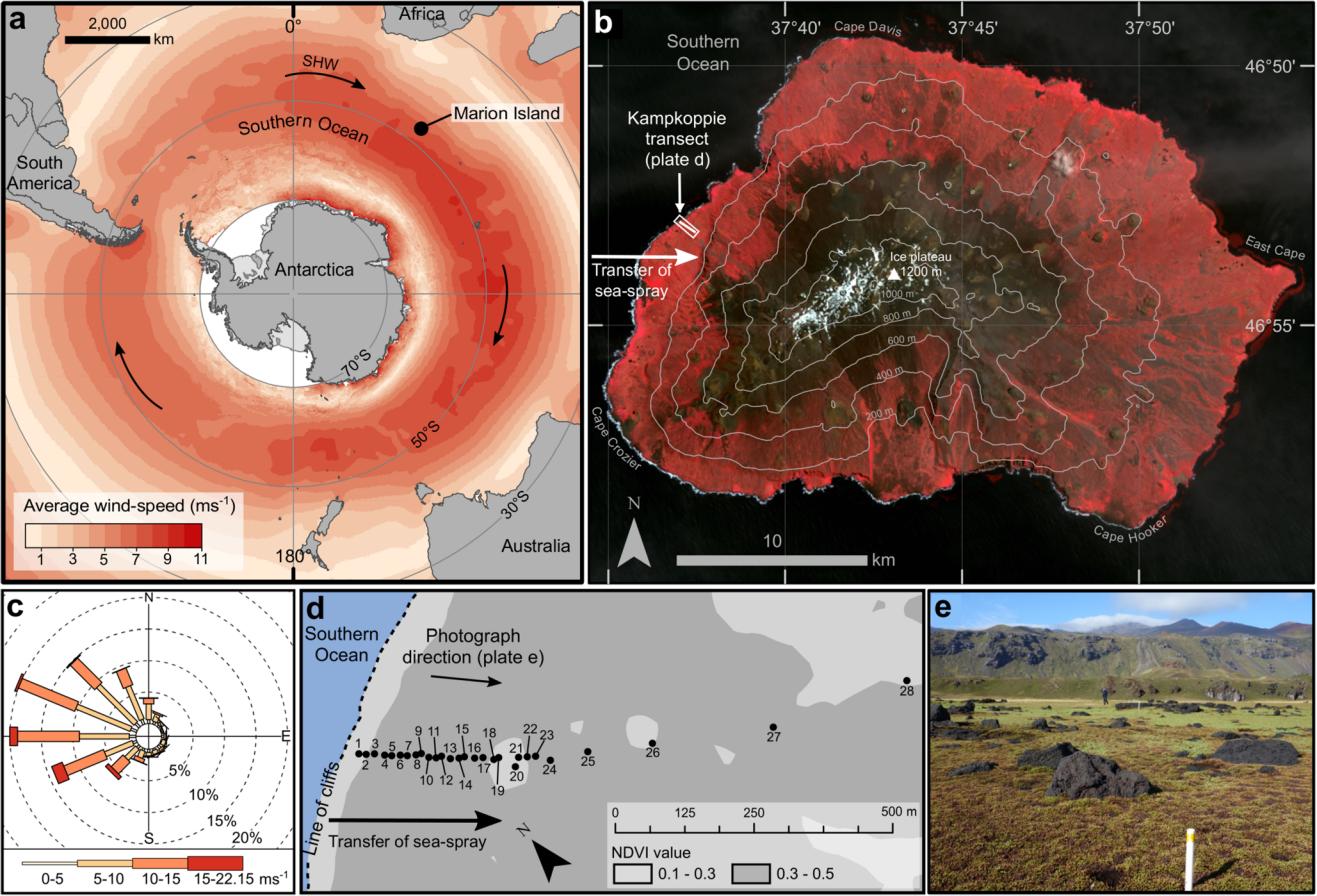
**a** Location of Marion Island in the Southern Indian Ocean province of sub-Antarctica and within the core belt of the Southern Hemisphere Westerly winds (SHW). Arrows indicate prevailing atmospheric circulation. Annual sea surface-level (10 m) mean wind speeds are based on NOAA blended high resolution (0.25 degree grid) vector data downloaded from (https://www.ncdc.noaa.gov/data-access/marineocean-data/blended-global/blended-sea-winds), and are calculated from monthly mean values for the period 1995–2005. **b** False-colour satellite image indicating vegetation cover (red shading) and the peatland at Kampkoppie where samples were collected. Image courtesy of the US Geological Survey (http://earthexplorer.usgs.gov/). **c** Wind-rose derived from daily NOAA blended sea-winds data (source as Fig. 1a) for the period 2008–2018, indicating the prevailing winds from the west-north-west. Wind strengths and frequency were calculated for wind directions at 22.5 degree intervals. **d** Locations of samples (marked 1–28) within the gradient of salt-enrichment at Kampkoppie. Normalised difference vegetation index (NDVI) values indicate minor changes in the vegetation cover at the peatland surface. **e** Photograph of the peatland surface looking east from the coast and showing the lack of topographic variability along the sampled gradient. Vegetation is characteristic of exposed coastal areas with a salt-spray complex of *Cotula plumosa* and *Crassula moschata* (foreground) and exhibits a distinct succession with distance inland. Palaeocliffs visible in the background mark the most inland extent of the sampling transect (Photograph source: Dominic Hodgson)

**Fig. 2:**
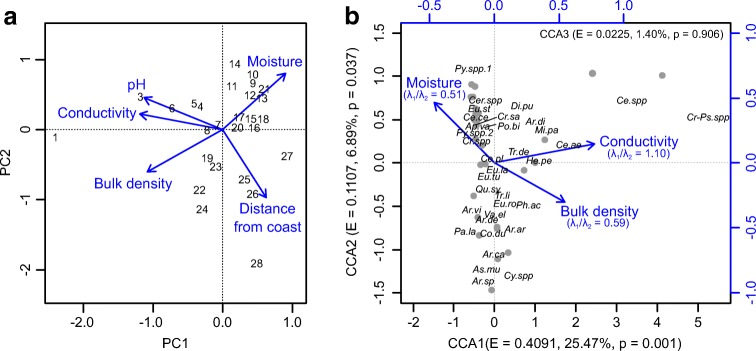
**a** Principal component (PC) analysis of environmental conditions represented in the sampled communities of Arcellinida and Euglyphida. Numbers indicate sample locations within the salinity gradient labelled sequentially with distance inland from the coastline (see Fig. 1d). High correlation between conductivity and pH of pore-water (*R*^2^ = 0.82, *p* < 0.001) and distance inland from the coast is indicative of oceanic salt-spray inputs, which cause a simultaneous increase in ionic strength and pH of the otherwise freshwater and acidic peatland ecosystem (i.e., both pH and conductivity act as proxies for salt-enrichment in this environment). **b** Canonical correspondence analysis (CCA) tri-plot representing the relationship between key micro-environmental variables and taxonomic assemblages. The ratio > 1 between the first constrained (λ_1_) and unconstrained axis (λ_2_) from a partial CCA run individually for each micro-environmental variable shows that conductivity is the main determinant of relative taxon abundance. pH and distance from the coast were removed from analysis due to their high correlation with conductivity. Eigenvalues (E) and percentage of variance in assemblage data explained by each axis is shown. Values of each environmental variable increase in the direction of the arrow. Abbreviations of individual taxa are given in supplementary table S1. Grey markers designate the distribution of samples. Full summaries of analysis are given in supplementary table S2 and S3

We recorded a diverse fauna of 34 taxa from 21 genera, including 17 taxa not previously identified on Marion Island (Supplementary figure S1; table S1). We also report the first known occurrence of *Quadrulella symmetrica* within the sub-, maritime-, or continental Antarctic region. Taxa belonging to the third major group of testate amoebae, Amphitrematidae (Labyrinthulids; SAR), were not present in these samples.

Marked differences in the AE fauna were observed between samples. Canonical correspondence analysis (CCA) indicated that measured conductivity is (or is linearly related to) the main determinant of assemblages (i.e. relative taxon abundance) in the sampled communities denoting a connection between AE fauna and magnitude of salt-enrichment (Fig. 2b). In total, conductivity accounted for ~ 48% of explained variance in the assemblages, and also explained the largest independent portion of variance after removing the contributions of confounding microhabitat variables (Supplementary table S4). Despite major differences in habitat, this relationship corroborates the role of salinity as a driver of assemblage changes suggested from observations of freshwater lakes exposed to salt addition of a comparable magnitude [12, 13].

Although the distribution of samples is skewed toward lower salinity conditions, tight clustering at the centre of CCA axis 1 indicates that saline conditions are sub-optimal for the majority of taxa. Accordingly, communities exposed to high concentrations of salt-spray deposition generally presented lower taxonomic-richness and evenness, implying that many taxa are selectively excluded by high salinity conditions (Fig. 3). Opportunistic generalist taxa from the genus *Centropyxis* [16] which have been suggested to exhibit moderately euryhaline ecology [13, 14] are a notable exception. These taxa dominated high salinity communities and possibly benefit from reduced competition caused by exclusion of other taxa (Supplementary figure S2).

**Fig. 3:**
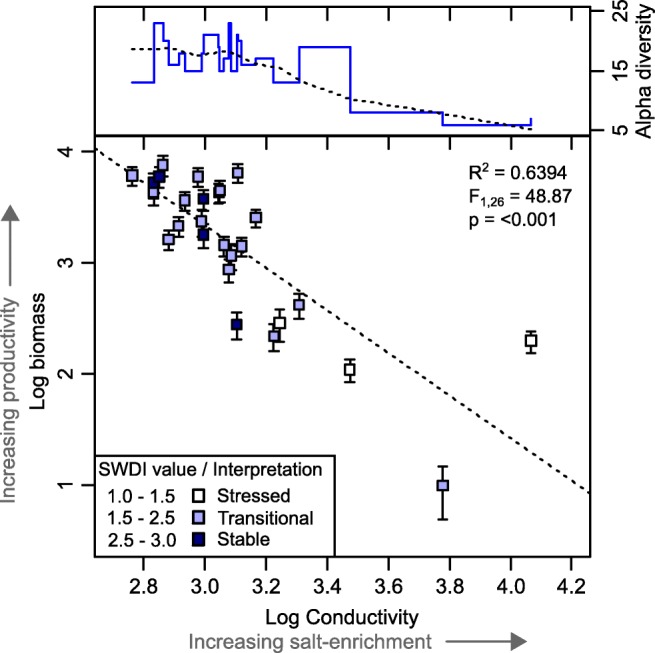
Relationship between pore-water conductivity and AE alpha-diversity, Shannon-Weaver diversity index (SWDI) and community biomass. Conductivity of pore-water was used as an indicator of salt-enrichment level in each community. Higher diversity, SWDI value and biomass are indicative of more productive AE communities. Interpretation of SWDI values indicate the favourability of environmental conditions for AE colonisation; healthy faunas in stable environments are characterised by high values, whereas low values represent faunas dominated by a small number of taxa under stressed environmental conditions [16, 27]. Conductivity (microsiemens/cm) and biomass (g C g dry-soil^−1^) were log_10_(x) transformed. Error bars represent uncertainty in community biomass calculated using upper and lower estimates of AE concentration and standardised values for taxon-specific biovolume (Supplementary methods)

To test whether salinity elicits quantifiable changes beyond the distribution of individual taxa, we examined variability in total biomass ([18, 19]; Supplementary methods). Biomass co-varied with alpha-diversity and denotes a major distinction between productive freshwater communities and depauperate, less productive salt-enriched communities (Fig. 3). Specifically, biomass and conductivity are linked by a highly significant, negative logarithmic relationship (*R*^2^ = 0.6394, *p* = < 0.001). Arcellinida and Euglyphida responded consistently (*R*^2^ = 0.6283/0.6234 respectively, both *p* = <0.001); although, as a more diverse taxonomic group, Arcellinida contributed a larger portion to total biomass. This relationship held when communities in samples exhibiting high conductivity values—i.e. > 2 millisiemens per centimetre (mS/cm)—were sequentially excluded (Supplementary figure S3). Our data therefore indicates that AE are highly sensitive even to small perturbations in salinity; for the least saline sample included in this study, an increase in microhabitat conductivity of only 0.25 mS/cm (from 0.58 to 0.83 mS/cm) was estimated to result in a 50% biomass reduction.

AE are important heterotrophs in peatlands [18, 20, 21] and consume a wide range of microbial prey (i.e. bacteria, other protozoa, fungi, algae [22]). Following this, it is possible that the observed variability in AE biomass reflects unmeasured differences in the availability of these prey microorganisms. To test this, we examined correlations between conductivity conditions and the prevalence of functional-traits linked to feeding ecology. However, no significant relationships were found to support this interpretation (Supplementary table S5). Additionally, we did not observe the presence of foraminifera which can coexist with AE in brackish environments [8, 14, 16] and may compete for resources. Abiotic factors, therefore, appear to be more influential than feeding interactions in governing the distribution and abundance of AE under these conditions, although more research is needed into potential variability in predation pressure.

The relationship between AE biomass and salinity was largely independent from patterns in species turnover. Variability in biomass was driven primarily by the abundance of individuals (population size) despite large differences in the sizes of the individual assemblage constituents (Supplementary table S6). Accordingly, population size was strongly correlated with conductivity (*R*^2^ = 0.6631, *p* < 0.001), which is consistent with observations linking reduced concentrations of AE to greater marine influence in coastal marshes [e.g. 6, 23].

AE taxa perform reproduction either (1) via asexual binary fission, (2) as a result of sexual life-cycles, or (3) as a combination of both strategies [24]. Test dimensions are set either during or shortly after reproduction and so it is assumed that cells maintain a fixed biovolume throughout their lifetime [but see 25]. Therefore, if both a constant rate of predation pressure and constant decay rate of empty tests is assumed, observed biomass is related directly to the rate of reproduction. Consequently, we suggest that reduced biomass is partly the result of an increased energetic cost associated with osmoregulation which is progressively traded-off against reproduction under increasing salinity. Most taxa appear not to be adapted to conditions where pore-water conductivity exceeds ~ 3.0 mS/cm which may mark the transition to a prevailing hypertonic environment (Fig. 3). This explanation linking the ionic strength of the AE microhabitat with community-level biomass is also consistent with a reduction in biomass observed after artificial nutrient addition to an Arctic fen-dwelling community [19].

The highly sensitive response of AE biomass, combined with patterns in the diversity and distribution of taxa, offer substantive potential for the use of these organisms as a bioindicator of environmental salinity. Application of these relationships to sub-fossil AE communities could considerably advance palaeoclimatology by providing, for the first time, millennial scale records of changes in atmospheric circulation recorded by salt-spray aerosol deposition onto coastal peatlands. Additionally, AE could play an important role in monitoring salinisation of coastal peatland ecosystems that are vulnerable to increased inundation under projected sea-level rise [see 26]. Biomass especially appears to offer a highly sensitive proxy for relative salt-enrichment, with the benefit of increased analytical efficiency over traditional approaches which require identification of individual taxa. More research should be conducted to confirm that comparable responses are observed under both experimental laboratory conditions and in other salt-stressed soil ecosystems.

Our results indicate for the first time that the effects of salt-enrichment on AE communities extend beyond previously documented changes in taxonomic composition to include strong effects on biomass production. AE offer potential as bioindicators of salinity with the capability to resolve low magnitude changes in salt-enrichment, such as deposition of wind-blown oceanic salt-spray.

## Electronic supplementary material

ESM 1 (PDF 880 kb)
