# Supplementary material for: Salt-Enrichment Impact on Biomass Production in a Natural Population of Peatland Dwelling Arcellinida and Euglyphida (Testate Amoebae)
Source: Microb Ecol. 2018 Dec 11;78(2):534–8. doi: 10.1007/s00248-018-1296-8 (PMC6647189; doi:10.1007/s00248-018-1296-8)
Supplement: Supplementary file 1 — (PDF 880 kb) [file 248_2018_1296_MOESM1_ESM.pdf]

Supplementary Information

Salt-Enrichment Impact on Biomass Production in a Natural Population of Peatland Dwelling Arcellinida and Euglyphida (Testate Amoebae)

Alex Whittle[1,2,*], Matthew J. Amesbury[1,3], Dan J. Charman[1], Dominic A. Hodgson[2,4], Bianca B. Perren[2], Stephen J. Roberts[2], Angela V. Gallego-Sala[1].

[1] Department of Geography, University of Exeter, Exeter, EX4 4RJ, UK.

[2] British Antarctic Survey, Natural Environment Research Council, High Cross, Madingley Road, Cambridge, CB3 0ET, UK.

[3] Environmental Change Research Unit (ECRU), Faculty of Biological and Environmental Sciences, University of Helsinki, Helsinki, Finland.

[4] Department of Geography, Durham University, Durham, DH1 3LE, UK.

*** Corresponding author:** aw424@exeter.ac.uk

Supplementary methods

Study site and salinity gradient.

Marion Island (46°54'S, 37°45'E) is an extremely isolated landmass of 290 km^2, located in the South Indian Ocean province of the sub-Antarctic biome (see Fig. 1 in main text). Climatic conditions are oceanic; mean annual temperature is 6.4°C, and mean annual precipitation is c.2000 mm (Le Roux & McGeoch, 2008a). Temperatures are stable with only 3.6°C difference between the warmest and coldest months, and a mean diurnal variability of 1.9°C (Smith, 2002). Southern Hemisphere westerly winds (SHW) are a dominant feature of the climate; mean annual wind-speed is 9.5 ms^{-1} (34.2 km hr^{-1}) (based on daily NOAA blended seawinds data for the period 2008-2018 (see Fig. 1 in main text for source)), and gale force winds (>18.3 m s^{-1}, 65.9 km hr^{-1}) occur more than 100 times per year (Gremmen et al. 1998) often lasting for 10 hours (Hedding et al. 2015).

Samples were collected during an expedition to Marion Island in April-May 2013. A transect aligned to the direction of the prevailing wind (i.e. East to West) was established across an area of low elevation coastal peatland at Kampkoppie on the west coast of the island (see Fig. 1 in main text). The transect stretched ~1 km inland of low sea-cliffs (~20 m above sea level) and spanned a coastal plateau with an altitudinal range of <50 m. No direct contact occurs between the peatland and ocean, although atmospheric transport and subsequent deposition of oceanic salt-spray is an important feature of coastal areas (e.g., Smith, 1978; Hänel & Chown, 1998; Le Roux & McGeoch, 2008b; Yeloff et al, 2007).

Peat forming vegetation at Kampkoppie consisted of a graminoid rich flora (e.g., *Agrostis magellanica, Uncinia compacta, Juncus scheuchzerioides*), and notably did not include *Sphagnum* mosses. The surface of the peatland is flat without a microtopography of hummocks and hollows which allowed the depth to water-table - a major influence on the distribution of Arcellinida and Euglyphida in other ecosystems – to be controlled.

Samples of the peatland surface (monoliths of 10 x 10 x 10 cm) were collected along the transect at 28 locations at 10 m intervals, with greater intervals for samples 24-28. Sample numbers were allocated sequentially (i.e. 1 = most coastal, 28 = furthest inland).

All samples were stored frozen and sealed within plastic bags prior to analysis. Conductivity and pH was recorded using a calibrated Hanna Instruments HI98129 meter from pore-water extracted by applying a compressive force to sub-samples of the surface 5 cm of each monolith. The relative level of salt-enrichment received by each sample was quantified by proxy of pore-water conductivity which is linearly related to salinity under these measurement conditions (Wagner et al, 2006). Physical properties of the substrate were measured in a separate sub-sample of 2 cm^3 (also from the surface 5 cm); bulk density was calculated by dividing the dry mass of each sample by its volume, and the change in sample mass after drying determined the moisture content (%).

Enumeration of Arcellinida and Euglyphida populations.

Tests (shells) of Arcellinida and Euglyphida were isolated from 1 cm^3 sub-samples collected from the surface (1 cm) of each monolith, and were concentrated for direct observation using a standard water-based protocol (Charman et al, 2000; Booth et al, 2010). One tablet of *Lycopodium* spores (Lund University; Batch number 1031) was added as an exotic marker in each sample (Stockmarr, 1971). Samples were boiled in 100 ml of de-ionised (DI) water for 10 minutes to disaggregate tests from the substrate, and allowed to cool to room temperature. The samples were then rinsed through sieves with DI water, and residues of the 15-300 µm fraction were retained for analysis. Supernatant water was removed by centrifuge (3000 rpm for 5 minutes).

Slides were prepared by diluting the residue with glycerol, and counts of taxon abundance were made at x200-400 magnification on a Zeiss AxioImager A1 light microscope. A minimum of two slides were analysed from each sample.

Counts included all individuals (i.e. living, encysted, and empty tests) to minimise possible assemblage bias caused by seasonal blooms (Barnett et al, 2013). A minimum of 200 individual tests were counted in each sample to detect subtle assemblage changes and to ensure that total diversity was accounted for (Payne & Mitchell, 2009). This threshold could not be reached for samples 2 and 3, where 12 and 50 individuals were observed respectively. A lower count total was considered sufficient because of the low diversity of taxa in the assemblage of sample 3 (Payne & Mitchell. 2009), and so these data were included in analysis. We also consider the low concentration of tests in sample 2 to be a valid result since the occurrence of *Lycopodium* spores rules out error in sample preparation, however these data were omitted from species-level analysis since the count total was deemed insufficient to accurately represent the taxonomic composition of the community.

Measurements of test dimensions were made using AxioVision software (version 4.8.2.0) coupled to a AxioCamHR3 camera. Tests are most commonly observed in broad lateral view during routine counting so measurements of test height (i.e. in the plane perpendicular to width and length) were made using a stereomicroscope. Literature values were used to supplement the data where it was not possible to obtain a sufficient number of measurements (Table S6).

Concentration of tests per cubic centimetre was calculated from the ratio of tests to *Lycopodium* spores, and converted to tests per dry gram using substrate bulk density (see Royles et al, 2016). Average biovolume of each taxon (Table S6) was calculated assuming standard geometric test shapes (Mitchell, 2004), and converted to biomass using the factor $1\ \mu m^3 = 1.1 \times 10^{-7}\ \mu g$ C (Mitchell, 2004). For each sample, total biomass was calculated by multiplying these values by taxon abundance. Error in biomass estimates was quantified by comparing maximum and minimum estimates, each based on upper (75%) and lower quartile (25%) ideal individual test dimensions and maximum and minimum test concentration values, respectively.

Shannon-Weaver diversity index values (Sageman & Bina, 1997) were calculated for each sample using the equation:

$$SWDI = -\sum_{i=1}^{S} \left(\frac{Xi}{Ni}\right) \times \ln\left(\frac{Xi}{Ni}\right)$$

Where S is richness of taxa (alpha-diversity), X_i is the abundance of each taxon, and N_i is the total abundance of Arcellinida and Euglyphida within the sample. We assumed that values; 2.5-3.5, represent stable environmental conditions, 1.5-2.5, transitional, and 0.1-1.5 are indicative of stressed conditions dominated by a small number of taxa (Patterson & Kumar, 2002).

Morphological trait values associated with feeding ecology were measured for each taxon (Table S5). Community weighted means for trait prevalence in each sample were calculated by multiplying these values by taxon abundance using the add-on package for R FD (Laliberté et al, 2014). Relationships between the community weighted mean value for each trait and conductivity were then assessed.

Taxonomy.

Identification of taxa was based on Ogden and Hedley (1980), Charman et al, (2000), Mazei and Tsyganov (2006), and Meisterfeld (2002a,b) with additional use of photographs and descriptions of southern hemisphere taxa (Fernández et al, 2015; Zapata & Fernández, 2008; van Bellen et al, 2014) and saltmarsh taxa (Charman et al, 2002; Gehrels et al, 2006). For standardisation with existing studies, identification used morphological features of the test, including; composition, shape, ornamentation, size and colour. We adopted a conservative approach to taxonomy to ensure that taxa represent biological species as closely as possible, and to produce an accurate estimation of diversity. Following Charman et al (2002), complexes of taxa (e.g., the genus *Centropyxis*) that are difficult to identify by test morphology were divided to the lowest possible level whilst maintaining clear, consistent and convenient morphological criteria. Microphotographs of all observed taxa are shown in Figure S1, and a list of taxa is given in Table S1.

Analysis of community assemblages.

All observed taxa were included in statistical analysis. Prior to analysis, assemblage counts were converted to relative abundance (percent) and square-root transformed to reduce the influence of dominant taxa (see Vincke et al, 2004). Detrended correspondence analysis (DCA) was used to estimate the overall gradient length of the assemblage data by determining whether species responses were primarily unimodal (gradient length > 2 standard deviations) or monotonic (gradient length < 2) (Legendre & Birks, 2012). Gradient lengths exceeded 3.0 standard deviations (3.07 for square-root transformed assemblage data), and therefore unimodal ordination methods were used to analyse the relationship between assemblages and corresponding environmental variables (ter Braak & Prentice, 1988).

Principal component analysis (PCA) was used to determine the major gradients in the environmental data. Prior to analysis environmental variables were checked for skewness, centred and standardised. Skewness of conductivity was reduced by applying a $\log_{10}$ transformation. Both conductivity and pH were shown to correlate with distance inland from the coast (R^2 = -0.45, p = 0.05, and R^2 = -0.56, p = 0.01 respectively), which confirms that the salinity gradient is produced by spatially variable deposition of oceanic salt-spray (i.e. levels decrease with distance inland from the coast). pH and conductivity therefore did not represent independent ecological signals in this dataset. Instead, both reflect salt-enrichment of an otherwise low-pH freshwater environment. Since conductivity is linearly related to salinity, unlike pH, it was retained for further analysis as an indicator of salt-enrichment level while pH was removed. Distance inland of coast was also removed from subsequent analysis since it is a synthetic (i.e. non-ecological) variable.

Canonical correspondence analysis (CCA) with Monte Carlo permutation tests was applied to assess the statistical significance of the measured microhabitat variables in explaining variations in assemblages between samples. Partial-CCA where each environmental variable was included as the only explanatory variable, against the assemblage dataset, was used to measure the significance of each environmental variable. Interaction between variables was estimated by sequentially including each as a co-variable in successive iterations (Table S4). To measure the strength of each environmental variable, in explaining changes in assemblages between samples, we calculated the ratio between the first constrained (CCA1) and first un-constrained (CA1) axis. A value > 1 indicates that the variable represents an important ecological gradient (Juggins, 2013).

All analyses were performed in R (version 3.4.1) (R core development team, 2017), and add-on package vegan (Oksanen et al, 2017).

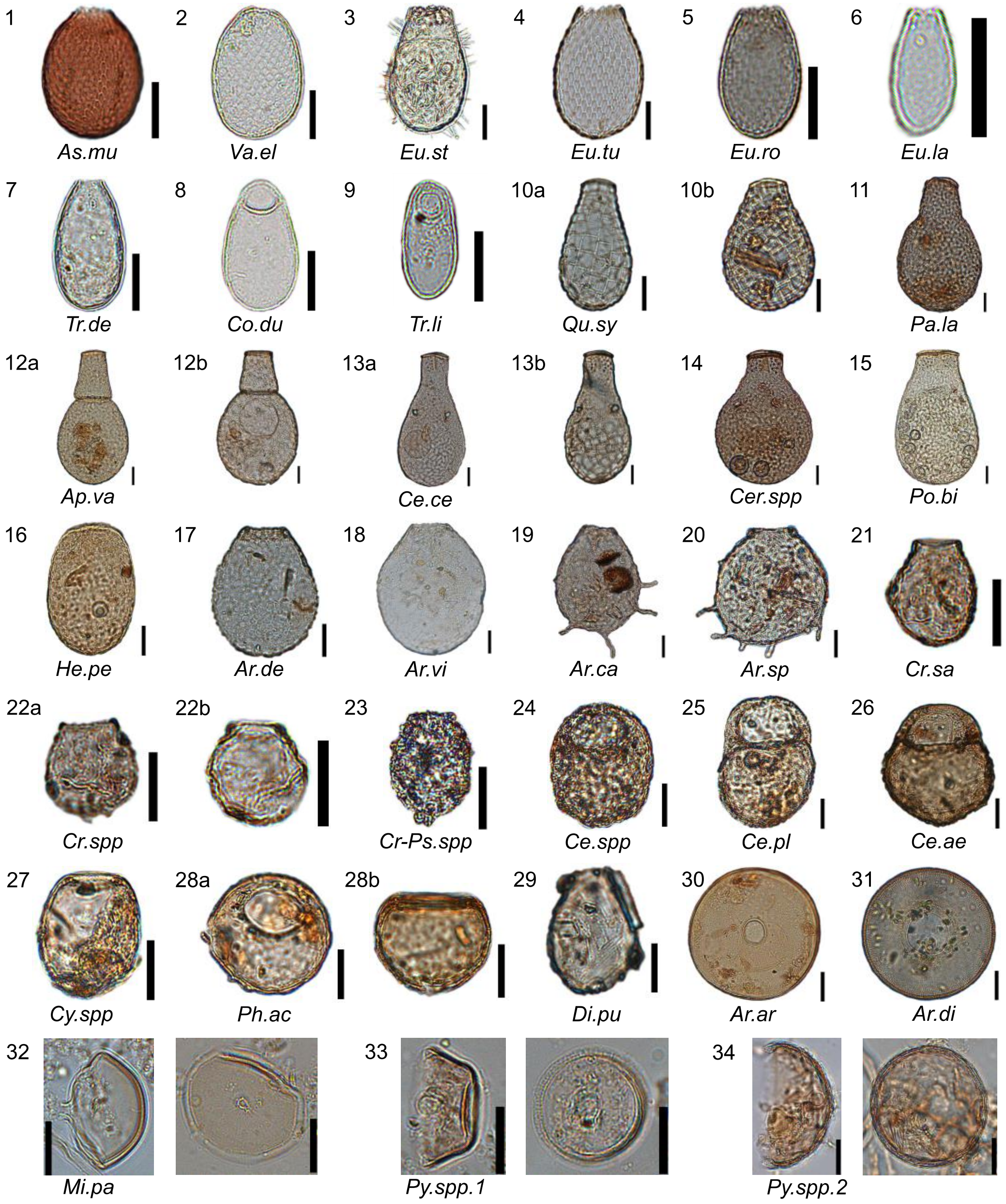

Figure S1 Arcellinida and Euglyphida taxa (collectively testate amoebae) identified in this study. Abbreviations for taxon name correspond to those used in Fig.2 (in main text). Reference numbers correspond to the full list of taxa given in Table S1. Images were obtained using a Zeiss AxioImager A1 light microscope coupled with an AxioCamHR3 camera. Scale bars represent 20 µm in all images.

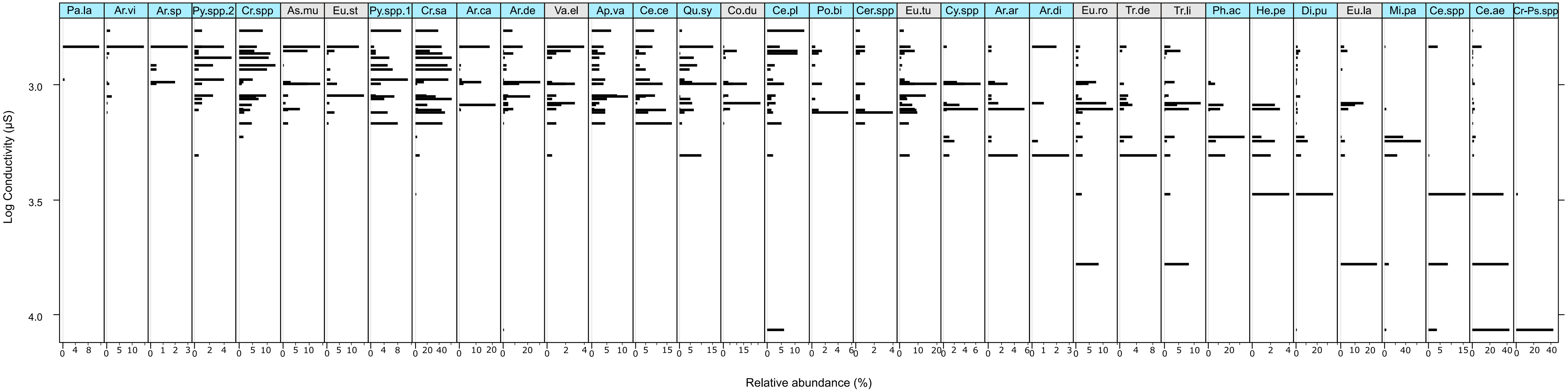

Figure S2 Percentage abundance of Arcellinida (blue shading) and Euglyphida (grey shading) taxa from the Kampkoppie peatland plotted against sample pore-water conductivity. Low conductivity values represent low levels of salt-enrichment. Taxa are ordered by conductivity optima calculated by weighted average. Taxon abbreviations refer to those given in Table S1.

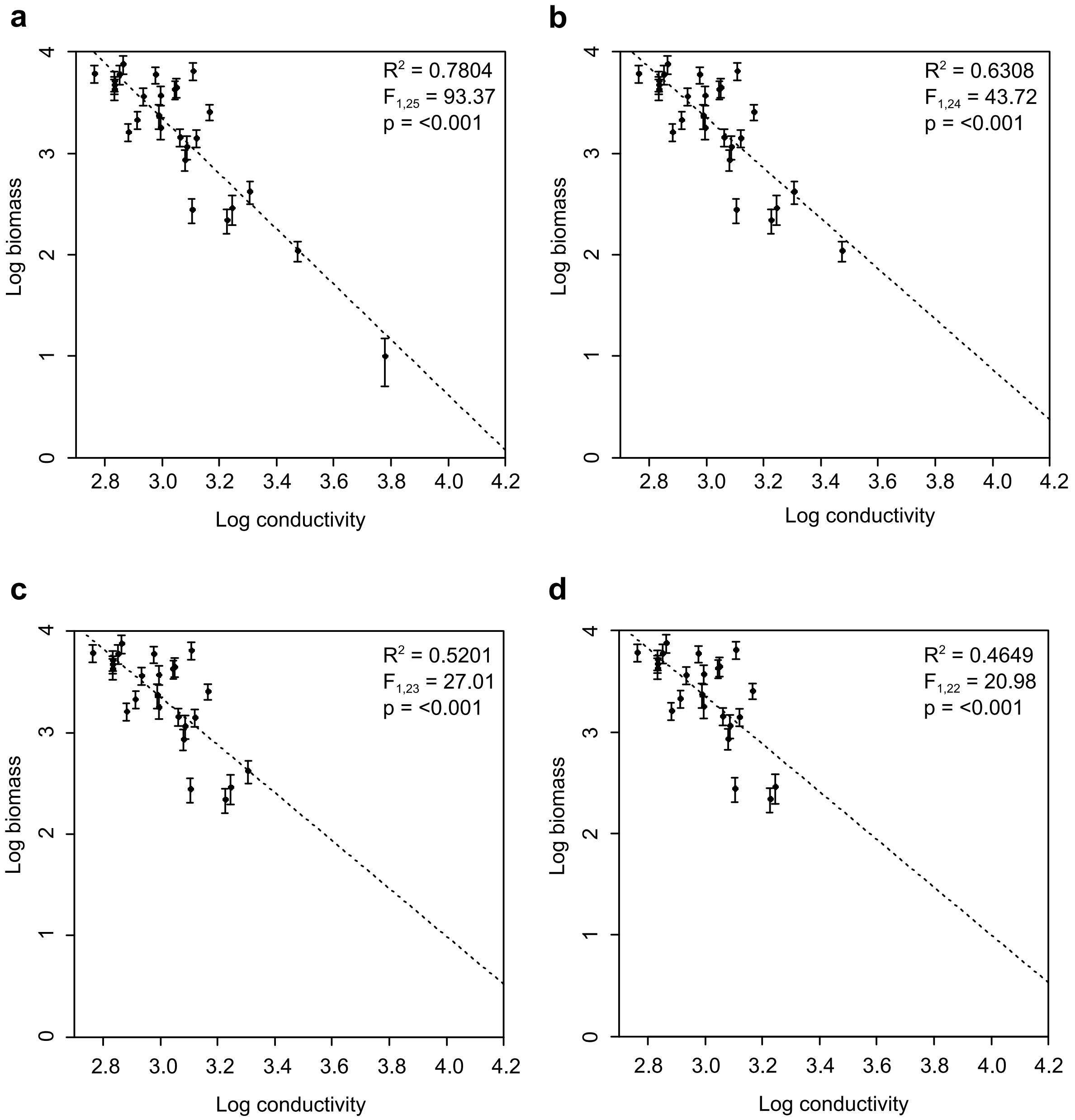

Figure S3 Relationship between pore-water conductivity (µS; microsiemens/cm) and testate amoebae biomass (g C g dry-soil^{-1}) after sequential removal of highly salt enriched samples (i.e. those with conductivity values > 2000 µS). Relationship for the range 580-6000 µS (a), 580-2980 µS (b), 580-2030 µS (c) and 580-1760 µS (d). Conductivity and biomass were $\log_{10}(x)$ transformed.

Table S1 List of all Arcellinida and Euglyphida taxa observed from the Kampkoppie peatland on Marion Island and corresponding abbreviations used in Fig. 2 (in main text). Taxonomy follows Krashevska et al (2016). One taxon could not be identified accurately to genus level and has been termed Cr-Ps.spp to reflect this uncertainty. 'Spp.' was used where taxa could only be confidently identified to the genus level, and 'type' for taxa exhibiting several morphotypes that possibly represent distinct species. Microphotographs of each taxa are shown in supplementary figure S1. • Indicates taxa not previously identified on Marion Island in the existing study of testate amoebae by Grospietsch (1971).

*Order / **Family	Taxon name / authority	Taxon abbreviation	Figure S1 reference number
*Arcellinida Kent 1880			
**Arcellidae Ehrenberg 1843			
	Arcella arenaria Greeff, 1866 •	Ar.ar	30
	Arcella discoides Ehrenberg, 1843 •	Ar.di	31
**Hyalospheniidae Schultze 1877			
	Quadrulella symmetrica (Wallich, 1863) Schulze, 1875 •	Qu.sy	10 a-b
	Apodera vas Certes, 1889	Ap.va	12 a-b
	Certesella certesi Penard, 1911	Ce.ce	13 a-b
	Certesella spp. •	Cer.spp	14
	Porosia bigibossa (Penard, 1890) Jung, 1942 •	Po.bi	15
	Padaungiella lageniformis (Penard, 1902) Lara and Todorov, 2012 •	Pa.la	11
**Microchlamyiidae Ogden 1985			
	Microchlamys patella (Claparède & Lachmann, 1859) Cockerell, 1911	Mi.pa	32
**Cryptodifflugiidae Jung 1942			
	Cryptodifflugia sacculus Penard, 1902	Cr.sa	21
	Cryptodifflugia spp. •	Cr.spp	22 a-b
	Cryptodifflugia-Pseudodifflugia spp. •	Cr-Ps.spp	23
**Centropyxidae Jung 1942			
	Centropyxis platystoma type (Penard, 1890) Deflandre, 1929	Ce.pl	25
	Centropyxis aerophila Deflandre, 1929	Ce.ae	26
	Centropyxis spp.	Ce.spp	24
**Phryganellidae Jung 1942			
	Phryganella acropodia (Hertwig and Lesser, 1874) Hopkinson, 1909	Ph.ac	28 a-b
**Trigonopyxidae Loeblich and Tappan 1964			
	Cyclopyxis spp. •	Cy.spp	27
** Incertae sedis Arcellinida			
	Argynnia dentistoma Penard, 1890	Ar.de	17
	Argynnia vitraea Penard, 1899 •	Ar.vi	18
	Argynnia caudata Leidy, 1879	Ar.ca	19
	Argynnia spicata Wailes, 1913 •	Ar.sp	20
	Difflugia pulex Penard, 1902 •	Di.pu	29
	Heleopera petricola Leidy, 1879 •	He.pe	16
	Pyxidicula spp.1 •	Py.spp1	33
	Pyxidicula spp.2 •	Py.spp2	34
* Euglyphida Copeland 1956			
** Euglyphidae Wallich 1864			
	Euglypha strigosa (Ehrenberg, 1871) Leidy, 1878	Eu.st	3
	Euglypha tuberculata Dujardin, 1841 •	Eu.tu	4
	Euglypha rotunda Wailes, 1915	Eu.ro	5
	Euglypha laevis (Ehrenberg, 1832) Perty, 1849	Eu.la	6
**Trinematidae Hoogenraad and De Groot 1940			
	Trinema lineare Penard, 1890	Tr.li	9
	Corythion dubium Taránek, 1881	Co.du	8
** Assulinidae Lara 2007			
	Assulina muscorum Greeff, 1888	As.mu	1
	Valkanovia elegans Schönborn, 1964 •	Va.el	2
** Incertae sedis euglyphid testate amoebae			
	Tracheleuglypha dentata Deflandre, 1928	Tr.de	7

Table S2 Principal Component (PC) analysis (a) axis scores, and (b) scores for each microhabitat variable.

		PC1	PC2	PC3	PC4	PC5	Sum of Eigenvalues
a)	Standard deviation	1.7142	1.1501	0.6342	0.4285	0.3911	5
	Eigenvalues	2.9383	1.3228	0.4022	0.1837	0.1530	
	Percentage of explained variance	58.77	26.46	8.05	3.67	3.06	
	Cumulative percentage of explained variance	-	85.22	93.27	96.94	100	
		PC1	PC2	PC3			
b)	Conductivity	-0.5243	0.1460	-0.4501			
	pH	-0.4970	0.3059	-0.4006			
	Moisture content	0.4091	0.5435	-0.3326			
	Bulk density	-0.4790	-0.4022	0.2112			
	Distance from coast	0.2850	-0.6542	-0.6940			

Table S3 Canonical correspondence analysis (CCA) of community assemblages and ecological microhabitat variables (pore-water conductivity, bulk density and moisture content).

Axes	CCA1	CCA2	CCA3	Sum of canonical Eigenvalues	Sum of Eigenvalues
Eigenvalues	0.4091	0.1107	0.0225	0.5423	1.6059
p-value	0.001	0.037	0.906		
Percentage of variance of species data explained	25.47	6.89	1.40		
Cumulative percentage variance of species data explained	-	32.36	33.76		

Table S4 a) Individual canonical correspondence analysis results. CCA1/CA1 is used to measure the strength of each explanatory variable in explaining changes in assemblages between samples, where a value >1 indicates that the corresponding variable represents an important ecological gradient (Juggins, 2013). b) Variance partitioning results. C – pore-water conductivity, M – moisture content, and BD – bulk density.

	Variable	Co-variable	CCA1	CCA1/ CA1	Sum of Eigenvalues	Variance explained by variable (%)	Interaction between variable and co-variable(s) (%)	*p*-value
a)	C	none	0.3892	1.10	1.6059	24.24	-	0.001
	M	none	0.2123	0.51		13.22	-	0.01
	BD	none	0.2224	0.59		13.85	-	0.001
b)	C	M	0.3066		1.6059	19.09	5.15	
		BD	0.2567			15.98	8.26	
		All	0.258			16.07	8.17	
						Total interaction:	**13.41**	
	M	BD	0.0619		1.6059	3.86	9.37	
		C	0.1297			8.08	5.15	
		All	0.0633			3.94	9.28	
						Total interaction:	**14.51**	
	BD	M	0.0720		1.6059	4.48	9.37	
		C	0.0899			5.60	8.26	
		All	0.0234			1.46	12.39	
						Total interaction:	**17.62**	

Table S5 Selected morphological traits associated with the feeding ecology of Arcellinida and Euglyphida. None of the relationships between the prevalence of traits and conductivity conditions were found to be statistically significant at the $p \leq 0.05$ level. CWM – community weighted mean, C – pore-water conductivity.

<table>
<tr><th colspan="2">Trait</th><th>Unit</th><th>Ecological interpretation</th><th>Reference</th><th>Correlation of trait CWM and C (R^2)</th><th>p-value</th></tr>
<tr><td colspan="2">Pseudopod type</td><td>Lobose or Filose</td><td>Filose taxa (Euglyphida) are mainly bacterivores and are considered to be r-strategists, whereas Lobose taxa (Arcellinida) are assumed to be K-strategists.</td><td>Fournier et al (2012)
Fournier et al (2016)</td><td>0.05</td><td>0.80</td></tr>
<tr><td>Test width</td><td rowspan="2">‘Body Size’</td><td rowspan="2">µm</td><td rowspan="2">Test size is assumed to relate to foraging characteristics, although the exact link between size and trophic level is unclear. Larger taxa appear to have longer generation times.</td><td rowspan="2">Lamentowicz et al (2015)
Fournier et al (2015)
Krashevska et al (2016)</td><td>-0.17</td><td>0.38</td></tr>
<tr><td>Test length</td><td>-0.24</td><td>0.22</td></tr>
<tr><td colspan="2">Aperture width</td><td>µm</td><td>Aperture dimensions are linked directly to the maximum size of food items which can be consumed. Taxa with a shell size >60 µm and aperture >15 µm are capable of consuming large prey items and hence it has been assumed that they occupy a high trophic level.</td><td>Lamentowicz et al (2015)
Fournier et al (2015)</td><td>-0.10</td><td>0.61</td></tr>
<tr><td colspan="2">Aperture width / Body length</td><td>Ratio</td><td>Low ratios are indicative of taxa which occupy a low trophic position (i.e. bacterivores and algivores), whereas a high ratio suggests a higher trophic position (i.e. taxa which predate other protists and micro-metazoan).</td><td>Jassey et al (2013)
Krashevska et al (2016)</td><td>-0.17</td><td>0.38</td></tr>
</table>

Table S6 Biovolume and biomass estimates for each taxon based on ideal individuals calculated from measurements of test dimensions. Bold values are derived from literature averages. Test shape classifications are based on the criteria defined by Mitchell (2004). Full names corresponding to the taxon codes are given in Table S1.

Taxon code / **Test shape classification**								Individual biovolume (μm^3)	Individual biomass (μgC g^{-1})	Literature data source
**Ovoid**	Test length (μm)			Test width (μm)			Test height (μm)			
	Mean	Std.Dev	*n*	Mean	Std.Dev	*n*				
Ap.va	151.80	9.79	*100*	80.12	7.90	*100*	**47.55**	385,500	0.0424	Mean calculated from data presented by Zapata and Fernández (2008)
Ar.ca	88.05	8.60	*62*	69.31	8.48	*62*	**49.50**	201,400	0.0222	Height based on *Ar.de* in Ogden and Hedley (1980)
Ar.de	88.17	9.02	*190*	73.47	11.55	*190*	**49.50**	213,800	0.0235	Ogden and Hedley (1980)
Ar.sp	90.81	8.13	*6*	79.92	5.25	*6*	**49.50**	239,500	0.0263	Height based on *Ar.de,* from Ogden and Hedley (1980)
Ar.vi	108.41	21.10	*14*	91.91	13.45	*14*	**75.00**	498,200	0.0548	Ogden and Hedley (1980)
As.mu	47.45	6.79	*92*	37.58	5.37	*91*	**20.00**	23,800	0.0026	Ogden and Hedley (1980)
Ce.ae	65.70	5.75	*81*	54.46	6.14	*78*	28.00	66,800	0.0073	
Ce.ce	138.95	8.18	*108*	75.17	6.53	*106*	49.87	347,300	0.0382	
Cer.spp	138.27	8.96	*11*	87.45	8.32	*11*	49.87	402,000	0.0442	
Ce.pl	86.00	8.05	*77*	62.82	7.30	*74*	48.95	176,300	0.0194	
Ce.spp	58.41	7.24	*14*	56.81	9.16	*14*	45.23	100,100	0.0110	
Co.du	38.56	5.64	*93*	25.39	3.25	*92*	**16.00**	10,400	0.0011	Cash and Hopkinson (1909)
Eu.la	26.28	5.21	*56*	13.78	3.22	*53*	6.89	1,700	0.0002	Test height estimated as width x 0.5 for all *Euglypha* spp.
Eu.ro	39.79	5.33	*83*	24.65	5.08	*83*	12.33	8,100	0.0009	
Eu.st	72.56	8.14	*45*	43.13	7.78	*43*	21.56	45,000	0.0050	-
Eu.tu	71.81	10.47	*194*	43.68	8.77	*175*	21.84	45,700	0.0050	-
He.pe	78.48	7.67	*16*	53.19	4.48	*16*	**45.00**	125,200	0.0138	Ogden and Hedley (1980)
Pa.la	139.54	8.43	*4*	82.71	7.76	*4*	**47.55**	365,900	0.0402	Based on *Ap.va*
Po.bi	145.48	10.33	*18*	80.40	8.93	*18*	49.87	388,900	0.0428	
Qu.sy	72.88	6.96	*94*	42.28	5.02	*87*	**29.61**	60,800	0.0067	
Va.el	44.08	6.02	*19*	29.41	4.12	*19*	**20.00**	17,300	0.0019	Based on *As.mu* following Royles et al. (2016)
**Saucer**	Test diameter (μm)			Test height (μm)						
	Mean	Std.Dev	*n*	Mean	Std.Dev	*n*				
Ar.ar	85.41	12.22	*30*	17.49	4.97	*5*		50,100	0.0055	
Ar.di	84.21	13.36	*7*	31.52	3.43	*2*		87,800	0.0097	
Mi.pa	42.41	6.89	*115*	14.71	4.28	*18*		10,400	0.0011	
Py.spp1	33.48	3.11	*34*	17.40	1.04	*2*		7,700	0.0008	
Py.spp2	70.52	7.30	*72*	43.18	15.15	*6*		84,300	0.0093	

**Cylindrical-ovoid**	Test length (µm)			Test diameter (µm)				
	Mean	Std.Dev	*n*	Mean	Std.Dev	*n*		
Di.pu	37.67	6.74	*73*	26.27	5.26	*71*	20,800	0.0023
Tr.de	47.13	5.36	*35*	26.61	2.88	*35*	26,700	0.0029
Tr.li	33.27	7.86	*52*	15.39	3.24	*40*	6,300	0.0007
**Hemispheric**	Test diameter (µm)							
	Mean	Std.Dev	*n*					
Cr.sa	29.48	3.66	*306*				5,200	0.0006
Cr.spp	27.76	2.50	*72*				5,000	0.0006
Cy.spp	42.26	6.41	*50*				19,800	0.0022
Ph.ac	40.74	4.45	*72*				16,100	0.0018
Cr-Ps.spp	32.77	3.04	*37*				6,300	0.0007

Supplementary references

Barnett, R. L., Charman, D. J., Gehrels, W. R., Saher, M. H. and Marshall, W. A. (2013) Testate amoebae as sea-level indicators in Northwestern Norway: developments in sample preparation and analysis. *Acta Protozoologica* **52(3)**: 115-128.

Booth, R. K., Lamentowicz, M. and Charman, D. J. (2010) Preparation and analysis of testate amoebae in peatland palaeoenvironmental studies. *Mires and Peat* **7**: 1-11.

Cash, J. and Hopkinson, J. (1909) The British freshwater Rhizopoda and Heliozoa. Volume II. Ray Society, London.

Charman, D. J., Hendon, D. and Woodland, W. A. (2000) The identification of testate amoebae (Protozoa: Rhizopoda) in peats. Quaternary Research Association, London.

Charman, D. J., Roe, H. M. and Gehrels, W. R. (2002) Modern distribution of saltmarsh testate amoebae: regional variability of zonation and response to environmental variables. *Journal of Quaternary Science* **17(5-6)**: 387-409.

Fernández, L. D., Lara, E. and Mitchell, E. A. D. (2015) Checklist, diversity and distribution of testate amoebae in Chile. *European Journal of Protistology* **51(5)**: 409-424.

Fournier, B., Coffey, E. E. D., van der Knaap, W. O., Fernández, L. D., Bobrov, A. and Mitchell, E. A. D. (2016) A legacy of human-induced ecosystem changes: spatial processes drive the taxonomic and functional diversities of testate amoebae in Sphagnum peatlands of the Galápagos. *Journal of Biogeography* **43(3)**: 533-543.

Fournier, B., Lara, E., Jassey, V. E. and Mitchell, E. A. D. (2015) Functional traits as a new approach for interpreting testate amoeba palaeo-records in peatlands and assessing the causes and consequences of past changes in species composition. *The Holocene* **25(9)**: 1375-1383.

Fournier, B., Malysheva, E., Mazei, Y., Moretti, M. and Mitchell, E. A. D. (2012) Toward the use of testate amoeba functional traits as indicator of floodplain restoration success. *European Journal of Soil Biology* **49**: 85-91.

Gehrels, W. R., Hendon, D. and Charman, D. J. (2006) Distribution of testate amoebae in salt marshes along the North American East Coast. *Journal of Foraminiferal Research* **36(3)**: 201-214.

Gremmen, N. J. M., Chown, S. L. and Marshall, D. J. (1998) Impact of introduced grass Agrostis stolonifera on vegetation and soil fauna communities at Marion Island, sub-Antarctic. *Biological Conservation* **85(3)**: 223-231.

Grospietsch, T. (1971) Beitrag zur ökologie der Testaceen Rhizopoden von Marion Island. In: Marion and Prince Edward Island, Report on the South African Biological and Geological Expedition 1965-1966, eds. Van Zinderen-Bakker, E. M., Winterbottom, J. M. and Dyer, R. A. Balkema, Cape Town: 411-423.

Hänel, C. and Chown, S. L. (1998) The impact of a small, alien invertebrate on a sub-Antarctic terrestrial ecosystem: Limnophyes minimus (Diptera, Chironomidae) at Marion Island. *Polar Biology* **20(2)**: 99-106.

Hedding, D. W., Nel, W. and Anderson, R. L. (2015) Aeolian processes and landforms in the sub-Antarctic: Preliminary observations from Marion Island. *Polar Research* **34(1)**: 26365.

Jassey, V. E. J., Chiapusio, G., Binet, P., Buttler, A., Laggoun-Défarge, F., Delarue, F., Bernard, N., Mitchell, E. A. D, Toussaint, M- L., Francez, A-J., Gilbert, D. (2013) Above-and belowground linkages in *Sphagnum* peatland: climate warming affects plant-microbial interactions. *Global Change Biology* **19(3)**: 811-823.

Juggins, S. (2013) Quantitative reconstructions in palaeoclimatology: new paradigm or sick science? *Quaternary Science Reviews* **64**: 20-32.

Krashevska, V., Klarner, B., Widyastuti, R., Maraun, M. and Scheu, S. (2016) Changes in structure and functioning of protist (testate amoebae) communities due to conversion of lowland rainforest into rubber and oil palm plantations. *PLoS ONE* **11(7)**: e0160179.doi:10.1371/journal.pone.0160179

Laliberté, E., Legendre, P. and Shipley, B. (2014) FD: measuring functional diversity from multiple traits, and other tools for functional ecology. R package version 1.0-12. https://cran.r-project.org/web/packages/FD/index.

Lamentowicz, M., Gałka, M., Lamentowicz, Ł., Obremska, M., Kühl, N., Lücke, A. and Jassey, V. E. (2015) Reconstructing climate change and ombrotrophic bog development during the last 4000 years in northern Poland using biotic proxies, stable isotopes and trait-based approach. *Palaeogeography, Palaeoclimatology, Palaeoecology* **418**: 261-277.

Le Roux, P. C. and McGeoch, M. A. (2008a) Changes in climate extremes, variability and signature on sub-Antarctic Marion Island. *Climatic Change* **86(3-4)**: 309-329.

Le Roux, P. C. and McGeoch, M. A. (2008b) Rapid range expansion and community reorganization in response to warming. *Global Change Biology* **14(12)**: 2950-2962.

Legendre, P. and Birks, H. J. B. (2012) From classical to canonical ordination. In: Birks, H. J. B., Lotter, A. F., Juggins, S., Smol, J. P. (Eds) Tracking environmental change using lake sediments Developments in Paleoenvironmental Research 5, Springer, Dordrecht.

Mazei, Y. and Tsyganov, A. (2006) Freshwater Testate Amoebae. KMK, Moscow (In Russian).

Meisterfeld, R. (2002a) Order Arcellinida Kent, 1880. In: Lee, J.J., Leedale, G.F., Bradbury, P. (Eds.), An illustrated guide to the protozoa, 2, second ed. Society of Protozoologists, Lawrence, Kansas, USA, pp. 827–860.

Meisterfeld, R. (2002b) Testate Amoebae with Filopodia. In: Lee, J. J., Leedale, G. F., Bradbury, P. (Eds.), An illustrated guide to the protozoa, 2, second ed. Society of Protozoologists, Lawrence, Kansas, USA, pp. 1054–1084.

Mitchell, E. A. D. (2004) Response of testate amoebae (Protozoa) to N and P fertilization in an Arctic wet sedge tundra. *Arctic, Antarctic, and Alpine Research* **36(1)**: 78-83.

Ogden, C. G. and Hedley, R. H. (1980) An atlas of freshwater testate amoebae. Oxford University Press, Oxford.

Oksanen, J., Blanchet, F. G., Friendly, M., Kindt, R., Legendre, P., McGlinn, D., Minchin, P. R., O'Hara, R. B., Simpson, G. L., Solymos, P., Stevens, M. H. H., Szoecs, E. and Wagner, H. (2017). vegan: Community Ecology Package. R package version 2.4-4. https://CRAN.R-project.org/package=vegan.

Patterson, R. T. and Kumar, A. (2002) A review of current testate rhizopod (thecamoebian) research in Canada. *Palaeogeography, Palaeoclimatology, Palaeoecology* **180(1-3)**: 225-251**.**

Payne, R. J. and Mitchell, E. A. D. (2009) How many is enough? Determining optimal count totals for ecological and palaeoecological studies of testate amoebae. *Journal of Paleolimnology* **42**: 483-495.

R Core Team (2017) R: A Language and Environment for Statistical Computing. R Foundation for Statistical Computing, Vienna, Austria, URL https://www.R-project.org/.

Royles, J., Amesbury, M. J., Roland, T. P., Jones, G. D., Convey, P., Griffiths, H., Hodgson, D. A. and Charman, D. J. (2016) Moss stable isotopes (carbon-13, oxygen-18) and testate amoebae reflect environmental inputs and microclimate along a latitudinal gradient on the Antarctic Peninsula. *Oecologia* **181(3)**: 931-945.

Sageman, B. B. and Bina, C.R. (1997) Diversity and species abundance patterns in Late Cenomanian black shale biofacies, Western Interior, USA. *Palaios* **12(5)**:449–466.

Smith, V. R. (1978) Animal-plant-soil nutrient relationships on Marion Island (Subantarctic). *Oecologia* **32(2)**: 239-253.

Smith, V. R. (2002) Climate change in the sub-Antarctic: an illustration from Marion Island. *Climatic Change* **52(3)**: 345-357.

Stockmarr, J. (1971) Tablets with spores used in absolute pollen analysis. *Pollen et Spores* **13**: 615–621.

ter Braak, C. J. F. and Prentice, I. C. (1988) A theory of gradient analysis. *Adv Ecol Res* **18**:271–317.

van Bellen, S., Mauquoy, D., Payne, R.J., Roland, T.P., Daley, T.J., Hughes, P.D., Loader, N.J., Street-Perrott, F.A., Rice, E.M. and Pancotto, V.A. (2014) Testate amoebae as a proxy for reconstructing Holocene water table dynamics in southern Patagonian peat bogs. *Journal of Quaternary Science*, **29(5)**: 463-474.

Vincke, S., Ledeganck, P., Beyens, L. and van de Vijver, B. (2004) Soil testate amoebae from sub-Antarctic Îles Crozet. *Antarctic Science* **16(2)**: 165-174.

Wagner, R. J., Boulger, R. W. Jr., Oblinger, C. J. and Smith, B. A. Guidelines and standard procedures for continuous water-quality monitors: station operation, record computation, and data reporting: U.S. Geological survey techniques and methods 1-D3. (2006) Available at: https://pubs.usgs.gov/tm/2006/tm1D3/. (Accessed: 6th June 2018).

Yeloff, D., Mauquoy, D., Barber, K., Way, S., van Geel, B. and Turney, C. S. M. (2007) Volcanic ash deposition and long-term vegetation change on subantarctic Marion Island. *Arctic, Antarctic and Alpine Research* **39(3)**: 500-511.

Zapata, J. and Fernández, L. (2008) Morphology and morphometry of *Apodera vas* (Certes, 1889) (Protozoa: Testacea) from two peatlands in Southern Chile. *Acta Protozoologica.* **47(4)**: 389-395.
